# Human Excretion of Bisphenol A: Blood, Urine, and Sweat (BUS) Study

**DOI:** 10.1155/2012/185731

**Published:** 2011-12-27

**Authors:** Stephen J. Genuis, Sanjay Beesoon, Detlef Birkholz, Rebecca A. Lobo

**Affiliations:** ^1^Faculty of Medicine, University of Alberta, 2935-66 Street, Edmonton, AB, Canada T6K 4C1; ^2^Department of Laboratory Medicine, University of Alberta, Edmonton, AB, Canada T6G 2B7; ^3^Environmental Division, ALS Laboratory Group, Edmonton AB, Canada T6E 5C1; ^4^Department of Family Medicine, University of Alberta, Edmonton, AB, Canada T6G 2C8

## Abstract

*Background*. Bisphenol A (BPA) is an ubiquitous chemical contaminant that has recently been associated with adverse effects on human health. There is incomplete understanding of BPA toxicokinetics, and there are no established interventions to eliminate this compound from the human body. Using 20 study participants, this study was designed to assess the relative concentration of BPA in three body fluids—blood, urine, and sweat—and to determine whether induced sweating may be a therapeutic intervention with potential to facilitate elimination of this compound. *Methods*. Blood, urine, and sweat were collected from 20 individuals (10 healthy participants and 10 participants with assorted health problems) and analyzed for various environmental toxicants including BPA. *Results*. BPA was found to differing degrees in each of blood, urine, and sweat. In 16 of 20 participants, BPA was identified in sweat, even in some individuals with no BPA detected in their serum or urine samples. *Conclusions*. Biomonitoring of BPA through blood and/or urine testing may underestimate the total body burden of this potential toxicant. Sweat analysis should be considered as an additional method for monitoring bioaccumulation of BPA in humans. Induced sweating appears to be a potential method for elimination of BPA.

## 1. Introduction

First synthesized in 1891 and with current production estimated at 4 billion kilograms each year globally [1], bisphenol A (BPA) is a multipurpose compound that is widely used in the modern industrial world. BPA was initially investigated for its potentially therapeutic estrogenic properties in the 1930s; when diethylstilbestrol (DES) was found to be more potent, however, BPA was temporarily cast aside. Its commercial value was reassessed in the 1950s with the introduction of BPA as a fundamental component in the manufacturing of some plastics. As its primary use currently, BPA is a key monomer in the production of the most common form of clear and shatter-proof polycarbonate plastic, but it has also been incorporated into a variety of everyday goods.

Questions regarding the safety and side effects of BPA began to emerge in the late 1990s when BPA was found to leech out of plastics and into experimental animal subjects, resulting in an increased incidence of chromosomal anomalies in offspring [2]. There has since been ongoing discussion in both scientific and political spheres about the potential for harm resulting from human BPA exposure and potential bioaccumulation. An overview of the literature regarding the effects of BPA on human health is provided, followed by a presentation of data from 20 subjects whose blood, urine, and sweat were tested for BPA. Results and discussion regarding BPA bioaccumulation and elimination are presented for consideration.

## 2. Background

Currently, BPA is most commonly found as a component in polycarbonates (~74% of total BPA produced) and in the production of epoxy resins (~20%). As well as being found in a myriad of products including plastic food and beverage containers (including baby and water bottles), BPA is also commonly found in various household appliances, electronics, sports safety equipment, adhesives, cash register receipts, medical devices, eyeglass lenses, water supply pipes, and many other products. It is also frequently used as an adjunct in the production of brominated flame retardants and brake fluid [1]. Moreover, BPA derivatives, such as bisphenol A-glycidyl methacrylate and bisphenol A-dimethacrylate, have recently been incorporated into the dental industry and used in dental fillings and sealants. The widespread use of this compound is receiving increasing scrutiny as concerns about BPA effects on human health have recently emerged.

The main mechanism by which the population is exposed to BPA is through leaching from plastic products. This results from either the release of unpolymerized monomers or the slow decay of polymer bonds in polycarbonates leading to monomer release into proximal foods and liquids. Occupational exposures are also present where plastics are burned and manufactured, and thus BPA may be inhaled by workers [3, 4]. An analysis of Chinese employees in factories where BPA and epoxy resins are produced, for example, revealed that over 90% of exposed workers have notable levels of BPA in their serum and urine [5].

A plethora of recent studies affirms that the majority of the population (91–99%) does indeed have detectable levels of BPA, but the level and the toxicological relevance of current exposure levels is a subject of intense academic and public health debate [6–13]. An extensive review conducted in 2007 concluded that BPA levels in human blood and/or urine are within the range shown to be dangerous in animals and are therefore likely to be biologically active in humans [6]. (As will be discussed, however, blood and urine testing may underestimate the full extent of exposure and bioaccumulation.) Conversely, an industry-sponsored literature review from 2008 declared that daily human consumption was far below dangerous levels and is therefore of minimal concern [7]. 

Sources of BPA ingestion may vary. In infants and children, baby and beverage bottles used by most individuals in the pediatric population provide ongoing daily sources of BPA [14–22]. Le et al. found that at room temperature, leaching of BPA occurred into the contained fluid, which increased 55-fold if boiling water was added [14]. Another study found that exposure levels increased not only with temperature, but also with repeated use of a container [15]. 

Other common sources of ingestion include foods stored in food cans, which are lined with BPA epoxy resin films to prevent corrosion [23–28], thermal printing paper commonly used in cash register receipts [29–31], and BPA containing dental composites and sealants [32–34]. Medical equipment is also raising concerns about BPA levels as a study of newborns found that those who regularly spent time in a neonatal intensive care unit had significantly higher serum BPA levels than the general population—thought to be due to exposure to plastics in medical devices [35]. Likewise, dialysis patients appear to have higher rates of exposure, which may be attributable to circulating solvents which expedite the leaching of BPA from polycarbonate hemodialysis equipment [36, 37].

When ingested, unconjugated BPA—the biologically active form of BPA—has historically been thought to be rapidly conjugated in the liver and then excreted through bile or urine, with a half life of approximately 5.3 hours [38–40]. This rapid excretion has been the basis of reassuring safety evaluations and declarations given by some public health authorities worldwide. However, within many tissues, particularly the lungs, livers and kidneys in rats, and the placenta of animals and humans, *β*-glucuronidase enzyme is present at detectable concentration. This enzyme is able to deconjugate BPA and thus release its active form again [41]. This is of great significance, as it is plausible that, in pregnancy, the conjugated form of BPA will circulate through the placenta, undergo deconjugation, and cause subsequent fetal exposure in utero. This may also result in bioaccumulation of some portion of BPA after exposure. In fact, recent evidence suggests that at low concentrations, while most plasma BPA (about 95%) is bound to serum proteins, BPA has lipophilic affinity with a fat: blood coefficient of 3.3 [42]. Furthermore, BPA appears to have a disproportionate affinity to fat in comparison to other tissues such as kidney, muscle, and other sites; “in fat, the accumulation of BPA was about three times higher than in other tissues” [42]. With evidence of potential bioaccumulation, BPA has the prospect of exerting ongoing metabolic effects.

### 2.1. Potential Implications of BPA Exposure

BPA is thought to wield its effects through endocrine disruption, epigenetic modification, cytokine release, and oxidative stress. When first discovered, BPA was investigated for its estrogenic properties, as it is thought to alter the synthesis of estradiol and testosterone and interfere with receptor binding [43, 44]. Consequently, exposure to BPA has been linked with a number of developmental and reproductive pathologies in both animal models and human subjects. These include abnormalities in reproductive organ function (irregular cycles, multiple ovarian cysts, reduction in primordial follicles [45–49]), placental dysfunction [50], increased incidence of miscarriage and neonatal mortality [50, 51], precocious puberty [52], and sexual dysfunction such as erectile dysfunction, decreased libido, and ejaculation difficulties [53–55]. Moreover, interference with the production and signaling of sex hormones has led to neurological impairment [56–60]. Synapse formation during development is regulated by estrogen and androgens; however with exposure to BPA, a recent study found that levels deemed safe by the US Environment Protection Agency, completely abolish the response of synapses to estrogen in the prefrontal cortex and hippocampus [61].

Epigenetic effects of BPA have been associated with an increased risk of cancer, particularly breast and prostate malignancies [62–69]. The exposure of breast epithelial cells to BPA was found to alter gene expression of 170 genes and increase their vulnerability to other carcinogens [62, 63]. Additionally, there was silencing of lysosomal-associated membrane protein 3, as occurs in ER*α*-positive breast cancer [62]. Similar effects have been seen with respect to prostatic disease, as exposure to BPA has been repeatedly shown to modify methylation of implicated genes [64–66].

Low dose BPA exposure at weaning during the perinatal period has also been found to increase adipogenesis in female animals [70]. As it is hypothesized that adult body weight may be programmed during early life, these results are noteworthy with regards to the childhood obesity pandemic and the action of endocrine disruptors as determinants of obesity [70]. BPA exposure appears to have widespread impact as it has also been linked by various researchers and studies throughout the world with a whole host of other health problems, including metabolic syndrome, obesity, non-insulin-dependent diabetes mellitus, allergies and asthma, ADHD, autism, cognitive decline, memory impairment, depression, and anxiety [71–92]. With the rise of sensitivity-related illness, there is also concern that BPA may be a determinant of this recently recognized causative mechanism of disease and source etiology of assorted clinical conditions [93] by stimulating the release of proinflammatory adipokines such as interleukin-6 (IL-6) and tumor necrosis factor alpha (TNF-alpha) from human adipose tissue [71]. 

As a result of all the emerging attention in the scientific literature, various governments have also embarked on research and policy decisions relating to BPA. In 2010, for example, the Minister of Health for the Canadian Government declared the results of a four-year study indicating, “The Government of Canada is a world leader in chemicals management. Our science indicated that Bisphenol A may be harmful to both human health and the environment” [94]. With emerging information of concern about BPA, the Canadian government became the first to prohibit the sale of BPA-containing polycarbonate baby bottles. France, Denmark and several American states have since implemented similar regulations.

### 2.2. Limitations of Toxicant Biomonitoring

It is often assumed that after exposure, BPA is rapidly metabolized to a hormonally inactive metabolite and efficiently excreted in toto from the body [95–97]. As a result, there has been little concern about bioaccumulation. The question arises, however, that if the compound is rapidly excreted without any accrual, why is there increasing evidence that BPA exposure is anything but innocuous and is able to cause potentially serious problems in animal and human organisms? While some believe that only unremitting ongoing exposure to BPA generates risk, concern has been raised that unrecognized bioaccumulation of some fraction of BPA may occur in some exposed individuals. How does one monitor to determine if toxicant compounds bioaccumulate and thus remain within the human organism? 

Throughout the world, blood and urine sampling are the general modalities used to biomonitor levels of most toxicant compounds including toxic elements, synthetic compounds, petrochemical compounds, biologic toxicants such as mycotoxins, and xenobiotics sometimes produced as byproducts from processing of parent compounds [98]. There is increasing evidence, however, that relying on blood and urine measurements as indicators of bioaccumulation can be very flawed [99]. Many compounds sequester in tissues and do not remain in blood; testing of whole blood or serum may miss toxicants which have exited the blood compartment and are being stored primarily in tissues such as bone, muscle, or adipose compartments. Levels of toxicant compounds can also rapidly fluctuate with changes in immediate status such as caloric restriction, level of hydration, underlying nutrient status, thermal changes, or exercise [98, 100]. 

Reliance on blood or urine testing for assessment of the body burden of many toxicants may thus be less than reliable clinically or for public health purposes. As a result, attempts to biomonitor toxicant levels by sampling other tissues and bodily excretions have been explored, including hair sampling, salivary testing, stool sampling, perspiration testing, breath analysis, provocation testing, and biopsies of adipose tissue through needle aspiration into fat pads under the skin. It is evident, however, that there are limitations with each of these approaches. Hair samples, for example may only reflect what has been in the blood stream for the last few weeks, while stool samples only assess what is being eliminated though fecal waste—these do not measure the body burden. As detailed toxicokinetics for many xenobiotic compounds are not fully understood, it is difficult to know which proportion of parent compounds and their metabolites accrue within various bodily compartments. 

Some researchers have recently commenced doing fat biopsies as a tool to biomonitor toxicant levels—this technique involves taking a sample of fat, usually from the abdominal or gluteal area and sending the sample for analysis. Recent evidence, however, confirms that toxicants sequester differently even within specific compartments such as adipose tissue; one adipose tissue site may display toxicant concentrations that are totally different than concentrations at another site [101]. So the toxicant concentration in brain adipose tissue, for example, may be very different than that found in breast or abdominal wall adipose tissue. 

In review, attempts to biomonitor the levels of toxicant compounds, including BPA, in humans using a single modality such as blood or urine are inadequate at best. This is a challenging realization as most population studies on toxicant compounds reported in the scientific literature as well as most ongoing biomonitoring research is based on blood or urine testing.

### 2.3. Study Objective

BPA exposure is generally assessed by measuring urine levels of this compound. In this research, we endeavor to determine the relative concentrations of BPA in blood, urine, and sweat. By assessing BPA levels in these three compartments, the possibility of identifying retained BPA will be explored as well as the potential for induced perspiration as a means to eradicate this compound.

## 3. Methods

### 3.1. Participant Recruitment

9 males and 11 females with mean ages 44.5 ± 14.4 years and 45.6 ± 10.3 years, respectively, were recruited to participate in the study after appropriate ethical approval from the Health Research Ethics Board of the University of Alberta. 10 participants were patients with various clinical conditions and 10 were otherwise healthy adults. Participants with health issues were recruited from the first author's clinical practice by invitation. Each participant in the study provided informed consent and volunteered to give one 200 mL random sample of blood, one sample of first morning urine, and one 100 mL sample of sweat. Demographic and clinical characteristics of all research participants are provided in Table 1.

### 3.2. Samples Collection

All blood samples were collected at one Dynalife laboratory site in Edmonton, Alberta, Canada through vacutainer blood collection equipment (BD Vacutainer, Franklin Lakes, NJ, 07417, USA) using 21 gauge stainless steel needles which were screwed into the “BD Vacutainer One-Use Holder” (REF 364815). The 10 mL glass vacutainer was directly inserted into the holder and into the back end of the needle. This process and the use of glass were used to preclude contamination. Blood was collected directly into plain 10 mL glass vacutainer tubes, allowed to clot, and spun down 30 minutes later. After serum was separated off, samples were picked up by ALS Laboratories (about 3 kilometres from the blood collection site) for storage pending analysis. When received at ALS, serum samples were transferred to 4-mL glass vials and stored in a freezer at −20°C, pending transfer to the analytical laboratory. We chose to analyze BPA in serum rather than in whole blood, based on the fact that matrix effect of serum is much lower than whole blood. 

For urine collection, participants were instructed to collect a first morning urine sample directly into a provided 500 mL glass jar container with Teflon-lined lid on the same day that blood samples were collected. Urine samples were delivered by the participants directly to Edmonton ALS Laboratories. Samples were transferred to 4-mL glass vials and stored in a freezer at −20°C, pending transfer. 

For sweat collection, participants were instructed to collect perspiration from any site on their body directly into the provided 500 mL glass jar container with Teflon-lined lid—by placing the jar against their prewashed skin when actively sweating or by using a stainless steel spatula against their skin to transfer perspiration directly into the glass jar. (Stainless steel—made up primarily of iron, chromium, and nickel—was chosen as it is the same material as the needles used in standard blood collections and is reported not to off-gas or leach at room or body temperature.) Excess of 100 mL of sweat was provided in all but one case. Each of the glass bottles used for sampling in this study was provided by ALS laboratories and had undergone extensive cleaning and rinsing. The containers were deemed appropriate for sweat collection with negligible risk of contamination: laboratory-grade phosphate-free detergent wash; acid rinse; multiple hot and cold deionized water rinses; oven-dried; capping and packing in quality controlled conditions. Sweat was collected within 1 week before or after doing the blood work. No specifications were given as to how long sweating had commenced before collection. 10 participants collected sweat inside an infrared sauna; 7 collected inside a regular steam sauna, and 3 collected during and immediately after exercise—no specific instruction was given regarding the type or location of exercise. Sweat was delivered by the participants directly to ALS laboratories. Samples were transferred to 4 mL glass vials and stored in a freezer at −20°C, pending analysis. No preservatives were used in the jars provided for sweat and urine collection, nor in the serum storage vials.

### 3.3. Analytical Methods

Human serum was analyzed for levels of bisphenol A (BPA) at ALS Canada following the general procedures presented by the Centres for Disease Control and Prevention [8, 102]. Briefly, samples were fortified with 12.5 nanograms of isotopically labelled phthalate metabolites, 50 nanograms of labeled bisphenol-A, 250 nanograms of 4-methylumbelliferone glucuronide, 300 microliters of ammonium acetate buffer (pH 6.5), and 10 microliters of *β*-glucuronidase (Escherichia coli K12, Roche Biomedical). The samples were mixed and incubated at 37°C overnight to allow for the deglucuronidation. 

Following enzymatic hydrolysis, a 20 uL aliquot of the sample is added to 70 uL of HPLC-grade water and 10 ng of labelled 4-methylumbelliferone to determine deglucuronidation efficiency (done once every 100 samples). The remaining sample is loaded onto a Zymark Rapid Trace Station for automated solid phase extraction (SPE). The 60 milligram/3 mL Oasis-HLB cartridges were conditioned with HPLC-grade methanol (2 mL) and 0.1 M formic acid (2 mL). The samples were diluted with 5 mL of 0.1 M formic acid and loaded onto the SPE cartridge at a rate of 1.0 mL/min. The cartridge was washed with water (1 mL) and 10% methanol in water (2 mL) at a flow rate of 1 mL/min. The samples were eluted with 1.0 mL of acetonitrile at a flow rate of 0.5 mL/min. The eluate was evaporated to dryness under a stream of dry nitrogen and the residue was resuspended in 85% methanol in water (200 microliters) and transferred to glass autosampler vials.

Quality control of the analysis was maintained by analysing a method blank (calf serum) and two spiked calf serum samples (20 ng/mL, all analytes) along with every 17 samples. The detection limit (0.2 ng/mL) was based upon our lower calibration standard (0.5 ng/mL) which gave an instrument signal to noise response of 3 : 1. Analysis was performed using an API 4000 liquid chromatograph/tandem mass spectrometer.

## 4. Results and Discussion

Demographic characteristics as well as results for each individual participant are summarized in Table 1. All concentrations are in nanograms per milliliter.

The fact that some subjects showed undetectable levels confirms that generalized contamination of these samples is not likely. Furthermore, the levels of BPA were similar to those recently published in studies from Italy and Greece [103] and are comparable with the serum levels found (0.79–7.12 ng/mL) in a recent study by Cobellis et al. in 2009 [104]. The rather low percentage detection among the serum samples in North America is hard to compare as (to our knowledge) there is only one study in the literature that documents BPA levels in blood of North Americans [105]. In that study, using the same extraction method as the methodology used in this study, the authors reported BPA levels in the range of <0.5 (detection limit) to 22.3 ng/mL in the blood plasma of 40 pregnant American women in the state of Michigan. However, the authors did not report in how many of these 40 women that BPA was detected above their current limit of detection of 0.5 ng/mL. In general, there is major variability in the range of concentrations of BPA detected in blood, and this may be explained by the fact that detection methods vary widely and the specific populations studied also vary considerably [106]. 

For the urine samples, the percentage detection in the current study (70%) is lower than that of the large scale Canadian biomonitoring study (90.7%) also known as the Canadian Health Measures Survey as reported by Bushnik et al. [10]. However, the geometric mean level of urine BPA in this study is generally higher than those in other biomonitoring studies in North America. Comparative levels for urine BPA as found in the literature are presented in Table 2. For example, Bushnik et al. reported a geometric mean of 1.15 ng/mL urine BPA in Canada for a sample size of 5462 [10], Calafat et al. found a value of 2.4 ng/mL for urine BPA among 950 American adults aged between 20 and 59 [9] and more recently Mendolia et al. reported on 375 American males with a geometric mean value of 1.50 ng/mL of urine BPA [107]. As far as sweat data is concerned, comparison across studies is impossible as to our knowledge this is the first study which attempts to quantify BPA in sweat.

**Figure 1:**
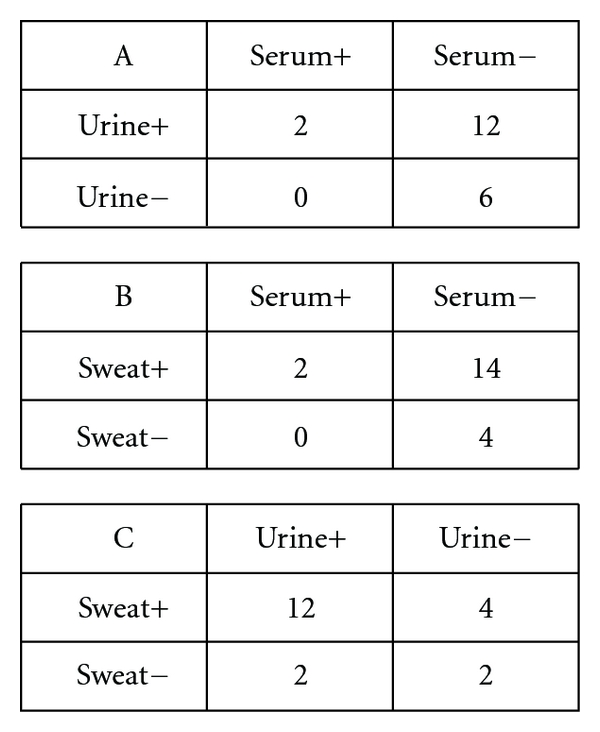
2 × 2 tables indicating the presence of BPA in specific body compartments. Each cell represents number of study participants.

One obvious qualitative interpretation of the data from the cohort of 20 study participants is that BPA is rarely detected in blood, and this is probably why most large scale biomonitoring studies, such as the NHANES (National Health and Nutrition Examination Survey) and CHMS (Canadian Health Measures Survey), use urine as the human sample of choice to determine exposure levels in populations. In an attempt to summarize the findings on the distribution of BPA in the 3 different body fluids, we give three 2 × 2 tables in Figure 1. As discussed earlier, the 2 discordant pairs urine+/serum− and sweat+/serum−, with 12 and 14 in their respective grids show clearly that serum is not the appropriate body fluid to test if BPA biomonitoring in humans is to be characterised. Although there seems to be high correlation between urine and sweat in terms of the presence/absence of BPA in these media, with 12 individuals in the urine+/sweat+ concordant pair, what is more surprising is that there are 4 individuals for which BPA was detected in sweat but undetectable in urine (Figure 1C).

In an attempt to compare the excretion efficiencies of urine and sweat for BPA, we calculated the ratio of BPA concentration in sweat versus urine for those 12 individuals who are in the urine+/sweat+ concordant pair. As shown in Table 1, with the exception of 2 individuals (participants 4 and 11) where urine concentration of BPA is slightly higher than in sweat, in general the ratio is higher than 1, suggesting that induced sweating may be an efficient method for eliminating BPA from the body. This is not surprising in the light of the findings of Csanády et al. whereby they found a preferential partitioning of BPA in adipose tissue compared to blood with a ratio of 3.3 [42]. This suggests that the BPA in blood which is then conjugated and excreted in the urine may only represent about one-third of the body burden of BPA. It is not surprising therefore that induced sweating in saunas can mobilise BPA in adipose tissue thus leading to enhanced excretion in sweat. Given that BPA in body fat is mostly unconjugated, further studies looking at the ratio of free BPA to conjugated BPA in sweat will help to confirm whether the BPA excreted in sweat comes from adipose tissue. Given that in normal circumstances the daily volume of urine is much higher than sweat, urine remains an important mode of elimination of BPA from the human body.

An important consideration in response to the results is why there are two participants with evidence of BPA in their urine with no positive level in their sweat. Presumably, the sweat level may be reflective of accrued toxicants in tissue, whereas urine may reflect in part at least recent exposure which the body is endeavoring to eliminate. It may be that BPA begins to bioaccumulate only after a threshold of exposure is reached or once the detoxification mechanisms of the organism are unable to completely eliminate the BPA load that presents after exposure. Further research is required in order to clarify, but this result may indicate that the two individuals have had some recent exposure, but no significant level of stockpiled toxicant. Similarly, the marked variation in the range of the sweat/urine ratio may once again be reflective of different phenomenon: the sweat results may represent transcutaneous excretion of accured BPA toxicant, while the urine results are perhaps reflecting recent BPA exposure in addition to some release of accrued BPA that the body is able to eliminate through renal mechanisms. It is also noteworthy that there was no statistically significant difference (*P*-value >0.05) in sweat BPA levels depending on the method of sweat collection whether through exercise, infrared sauna, or regular sauna. 

Although this study sheds light on the importance of sweat as a pathway of elimination for BPA from the body, it has some limitations. First, given the small study size (*n* = 20) it is not possible to extrapolate the findings to the general population. Secondly, samples were analysed for total BPA, instead of unconjugated BPA and conjugated BPA separately. Thirdly, other rare forms of BPA such as chlorinated and sulfated BPA were not analysed for. 

## 5. Conclusion 

As a result of increased scrutiny of health sequelae associated with human BPA exposure, it is apparent that this endocrine-disrupting compound has potentially negative consequences for the human organism. With new evidence for the possibility of BPA accrual within the body, interventions to facilitate elimination of this toxic compound have clinical relevance with regards to the prevention and treatment of adverse outcomes associated with BPA bioaccumulation. The results of this study suggest that: (i) Sweat testing may be an additional tool for BPA bio-monitoring; and (ii) Induced sweating appears to be a clinically useful tool to facilitate the release of BPA through the skin in order to eliminate this toxicant from the human body.

**Table:** 

	

Notable findings and implications of data from this study	

(i) BPA is excreted in sweat	
(ii) Sweat BPA concentrations are consistently much higher than urine	
(iii) Only 2/20 participants had BPA in serum, while 16/20 had BPA in sweat	
(iv) The data suggests that BPA likely bioaccumulates to some degree in humans	
(v) The data suggests that BPA retained in tissues (likely adipose) excretes via sweat	
(vi) The finding in some individuals that little or no BPA is excreted in urine while considerable levels are found in sweat suggests that current biomonitoring via serum (as done in Europe) or urine (as done in North America) may not provide a reliable indication of the BPA toxicant burden	
(vii) With the recognition that BPA has the potential for hormonal dysregulation, the significance of accrued BPA remains to be conclusively elucidated	

	

##  Conflict of Interests

There is no conflict of interests. No funding has been received for any part of this work.

## Figures and Tables

**Table 1:** Participant results for BPA in three body compartments: serum, urine, and sweat.

Participant	Gender	Age	Clinical diagnosis	Serum conc.	Urine conc.	Sweat conc.	Sweat/urine ratio	Technique used for sweat collection
1	M	61	Diabetes, obesity, hypertension	0	4	82	20.5	Exercise
2	F	40	Rheumatoid arthritis	0	22	24	1.1	Steam sauna
3	M	38	Addiction disorder	0	20	22	1.1	Steam sauna
4	F	25	Bipolar disorder	0	40	22	0.6	Steam sauna
5	F	47	Lymphoma	0	10	24	2.4	Steam sauna
6	F	43	Fibromyalgia	0	32	0	n/a	Steam sauna
7	F	48	Depression	0	0	16	n/a	Steam sauna
8	F	40	Chronic fatigue	0	0	22	n/a	Infrared sauna
9	F	68	Diabetes, fatigue, obesity	0	0	10	n/a	Steam sauna
10	M	49	Chronic pain, cognitive decline	0	8	10	1.3	Exercise
11	M	53	Healthy	10	32	20	0.6	Exercise
12	M	23	Healthy	0	30	46	1.5	Infrared sauna
13	M	21	Healthy	30	4	10	2.5	Infrared sauna
14	F	47	Healthy	0	8	12	1.5	Infrared sauna
15	M	53	Healthy	0	4	35	8.8	Infrared sauna
16	F	43	Healthy	0	0	12	n/a	Infrared sauna
17	F	51	Healthy	0	0	0	n/a	Infrared sauna
18	M	46	Healthy	0	42	0	n/a	Infrared sauna
19	M	57	Healthy	0	0	0	n/a	Infrared sauna
20	F	50	Healthy	0	8	22	2.8	Infrared sauna

**Table 2:** Comparison of urine BPA levels across published studies.

Urine levels of BPA (ng/mL)-comparison across studies							
Study	Location	Detection method	DL (ng/mL)	Participants	Detection(%)	AM/GM/Median	Range
This study	Canada	LC-MS	0.2	20 adults	70	AM:13 Median 8	0–42

Bushnik et al. [10]	Canada	GC-MS	0.2	5462 (age 6–79)	90.7	GM 1.16 (1.08–1.24)	N/A

Calafat et al. [9]	USA	LC-MS	0.4	950 adults	N/A	GM 2.4	N/A

Calafat et al. [8]	USA	GC-MS	0.1	394 adults	95	GM 1.33 Median 1.28	0.1–5.18 (*95th Centile*)

Moors et al. [108]	Germany	GC-MS	3	15 adults	60		ND-55

Mendiola et al. [107]	USA	LC-MS	0.4	375 males	90	GM 1.50	<0.4–6.5 *(95th Centile) *

DL: detection limit; AM: arithmetic mean; GM: geometric mean; ND: non detect; N/A: not available.

LC-MS: liquid chromatography-mass spectrometry.

GC-MS: gas chromatography-mass spectrometry.
